# Kojève’s silence: science and the end of history

**DOI:** 10.1007/s11007-026-09739-0

**Published:** 2026-05-12

**Authors:** Ties van Gemert

**Affiliations:** Department of Philosophy, Tilburg University, Tilburg, Netherlands

**Keywords:** Kojève, Hegel, Quantum physics, Niels Bohr, Werner Heisenberg

## Abstract

This paper argues that in his early writings on modern physics, Alexandre Kojève develops a critique of classical metaphysics that compromises his interpretation of Hegel. I aim to show that Kojève’s rejection of the classical conception of Being—as a spatially homogeneous uni-totality—renders the end of history impossible. I will contend that Kojève’s attempt to reconcile this tension through a distinction between knowledge and truth—asserting both the incompleteness of scientific knowledge andthe existence of an omniscient Wise Man—is ultimately untenable. In post-historicaltime, there can be no dualism of science and philosophy: the end of history implies—and therefore would coincide with—the end of science. The end of history not being possible without the end of science, the errors committed by men endure indefinitely.

## Introduction

In his only recently published book *Zum Problem einer diskreten »Welt«* (1929/2023), Kojève observes that with Werner Heisenberg’s formulation of the uncertainty principle, physics became modern —that is, it became aware of the subject—with indeterminacy now occupying a decisive place within this science, and the idea of a determinate world being annihilated: “Since 1926, physics is ‘for itself’, what is it ‘in itself’; the newest physics is a physics that understands itself (Hegel).”^1^ This statement on the relation between the uncertainty principle and Hegel’s conception of self-consciousness encapsulates the nucleus of this paper. Its objective is to show that in his early writings, Kojève develops a critique of classical metaphysics that compromises his interpretation of Hegel. The indeterminacy within the real disclosed by modern physics precludes the possibility of absolute knowledge.

The paper is divided into three parts. First, I set out Kojève’s conception of the worldview [*Weltanschauung*] of classical physics. Through a rearticulation of Laplace’s demon, he delineates the ambiguous place of subjectivity in classical physics. Next, I discuss Kojève’s account of the advent of modernity. In his book on Kant, Kojève argues that it is only with the introduction of the mode of the *as if* that the subject became recognized. During the nineteenth century, this subject became incorporated into physics as scientists came to understand that the idea of the determinate world could only be a regulative idea. In the third part, I turn to Kojève’s reflections on the subject of modern physics, reconstructing his understanding of Niels Bohr’s interpretation of quantum physics, as expressed in his commitment to an ontological dualism of observing and observed system.^2^ During his lectures on Hegel, Kojève reconceptualizes this dualism as a distinction between human being and natural being—or, following his interpretation of Heidegger, *Dasein* and *Vorhandensein*—insisting that this ontological difference is constitutive of the struggle required to institute the universal, homogenous state.^3^

The aim of this paper is to demonstrate that Kojève’s renunciation of the worldview of classical physics—that is, the conception of Being as a spatial-homogenous uni-totality—implies the impossibility of the end of history. Modern physics as exemplified in Heisenberg’s uncertainty principle reveals an irreducible duality within the real, occluding the possibility of reducing the human being to the natural being—neither the subject nor time will ever cease to exist. In the conclusion, I will argue that Kojève’s attempt to resolve this tension through a distinction between knowledge and truth—his commitment to, on the one hand, the incompleteness of scientific knowledge, and on the other hand, the existence of the omniscient Wise Man—is untenable. There can be no dualism of science and philosophy in post-historical time: the end of history implies—and therefore would coincide with—the end of science.

## The concept is eternity

In *The Idea of Determinism*, Kojève addresses the revolutionary developments in physics during the first decades of the twentieth-century. Rather than engaging in a philosophical discussion of the latest results, he takes their validity for granted. He does not think it is up to the philosopher to dispute results that are accepted by the majority of physicists, it is up “to physical science itself to decide.”^4^ Instead, the task that Kojève sets himself is a “logical elaboration’” of the spontaneous philosophy of the scientists: “[t]he philosophy of the scientists is not always very happy; but, if it is not, it is the philosopher who is responsible for it.”^5^ Methodologically, the philosopher’s work is immanent to the development of science. Their task is to explicate the commitments of a scientific worldview.

With deceptive humbleness, Kojève remarks that he will limit his focus to one aspect of the epistemological rupture, that is, the passage from the conception of the world as determinate to that of an indeterminate world. Evidently, this break is no trifling matter. As Kojève makes clear, it shatters our belief that the world can ever be known in its totality, forcing us to abandon the idea that “Being is essentially immutable in itself and eternally identical with itself.”^6^ At long last, so Kojève seems to allude, modern physics has annihilated the ideal of classical metaphysics—characterized in the opening paragraph of his study of Vladimir Solovyov as “a complete, homogenous and ordered whole to which may be applied the title ‘system of philosophy.’”^7^ As usual, Kojève puts himself in the position of an observer, with the intention of explicating the contradictions of classical physics, attempting “ to study its external ‘form’ by using the ‘behaviorist’ method.”^8^ His objective is to demonstrate that the idea of a determinate world was ambiguous from the time of its conception, that it contained tensions that have merely intensified. As he puts it, “[o]ur critique of the classical idea of determinism is, or at least intends to be, an immanent critique.”^9^ According to Kojève, these tensions have their origin in the preclusion of the subject of science: “[c]lassical physics had not sufficiently meditated on the idea of experience, and in particular it had not asked the question concerning the subject of experiments and its interaction with the object.”^10^ In his early writings, Kojève elaborates the philosophical-historical thesis that in the third decade of the twentieth century, physics as a science became aware of the subject: “the contribution of modern physics lies in the fact that it introduces a physical subject.”^11^ No longer aspiring to grasp the object as it is in-itself, as a being independent of the thought that describes it, it now finally incorporates the subject. Concisely put, modern physics has become self-consciousness, its being-in-itself has become a being-for-itself, “the ‘difference’ is the awareness of being, the givenness of the ‘difference’ is the consciousness of this consciousness, i.e., self-consciousness.”^12^ From Kojève’s perspective, Bohr’s interpretation of quantum physics epitomizes this expression of Hegel’s conception of self-consciousness. The subject of classical physics and the object of modern physics—these two opposed but inseparable worlds embodied in the observing and the observed system—are complementary yet cannot be unified without contradiction. In the first chapter of *The Idea of Determinism*, Kojève—referring to the early work of the founding father of the Vienna Circle, Moritz Schlick—paints for us with rough brushstrokes the outlines of the worldview of classical physics:

the idea of classical determinism generally took the form of the principle called < the principle of causality > : in the physical world nothing is fortuitous, everything is predictable; every phenomenon has a cause which necessarily precedes it, so that by knowing the cause we thereby know the effect; nothing is lost, nothing is created, the cause is preserved in the effect.^13^

The three principal commitments of the worldview of classical physics are succinctly captured as (1) the universality of the principle of causality, (2) the global validity of the law of conservation, and (3) the possibility that the subject can become omniscient, capable of complete and precise prediction. Explicating the principle of causality, Kojève again distinguishes three elements, namely (1) “the cause produces its effect (efficacy),” (2) “in knowing the ‘cause,’ we know the ‘effect’ (because the first is always followed by the second) (legality)” and (3) “ the cause is preserved in the effect (identity or Meyersonian ‘ causality’).”^14^ In what follows, he defines efficacy as “ a causal relation in the real” between two events A and B, where A has a “force” that “acts” on B, thereby producing B.^15^ In classical physics, Kojève claims, the relation between A and B is not to be understood as a correlation discerned by a subject (as in Ernst Mach’s phenomenalism), but as a structure of the real—efficacy implies metaphysical realism. This element of the principle of causality instantiates the commitment of classical physics to describe the real as a being-in-itself. For the idea of the determinate world of classical physics, the second element of causality is decisive. As Kojève puts it, “ in speaking of “determinism,” it is not the notion of causality strictly speaking but that of *legality* that we will mainly have to deal with in the following.”^16^ Referring to the Second Analogy of Experience in the first Critique, he argues that Kant expresses this principle in his first formulation of the principle of temporal sequence according to the law of causality: “[e]verything that happens [*geschiet*] presupposes something upon which [*worauf*] it follows according to a rule.”^17^ If every effect can be predicted by universal and necessary laws, and we know all such laws of the universe, we can foresee— i n detail and with precision—all that will happen. If Kant’s principle is valid, the phenomenal world has to be conceived of as strictly and completely determined.

The third element of the principle of causality is left largely undefined by Kojève. He offhandedly refers to it as “Meyersonian ‘causality’” but also designates it as the “ spirit” of classical physics.^18^ In his *Identité et réalité* (1908), Émile Meyerson had formulated a realist interpretation of the principle of causality, arguing that that the spontaneous philosophy of classical physics was inherently metaphysical, its aim being to *explain* the world by substituting for the diversity of reality abstract identities such as atoms, electrons, or phlogiston.^19^ By identifying cause and effect, showing that the latter is contained in the former, that there is a continuity between antecedent and consequent, classical physics spatialized time. To Meyerson, classical physics is the intellect’s tendency to geometrize the real, to reduce the heterogeneity of the world to one immutable, homogenous Being, while the real itself persists as an irrational negativity, an unknowable thing-in-itself.

According to Kojève, the ideal of classical physics delineated by Meyerson is epitomized in the second principle.^20^ Postulating the global validity of the law of conservation entails conceiving of the world as one object in which everything is contained. A uni-totality, which is “*one* because total and total because *one*.”^21^ In the terms of the classical physicist, the world is conceived as a closed, isolated system. It has no exterior: “if therefore there are things which exist outside this world, they cannot, by definition, have any kind of influence on it […] ‘the world taken in its entirety’ is a unique physical system, strictly isolated.”^22^ None of the laws governing the physical system allow for exception or change. All diversity is excluded, and nothing new can emerge. In the metaphysical terms employed by Kojève in chapter five of his lectures on Hegel, the Being of the classical physicist is One and because it is One, it is a Totality, thereby excluding the possibility of non-being and change.

In the world of classical physics, everything is encompassed as if in an eternal presence: “(classical) physics does not know […] finitude and [this] inevitably leads to the denial of change in the becoming of being and to the integration of everything in a single space […] [t]his tendency of (classical) physics is excellently shown by Meyerson .”^23^ As Kojève notes, Meyerson’s formulation of the principle of causality—“the same ‘causes’ always have the same ‘effects’”—collapses into a tautology, for either the causes and effects are identical or we deny that they are the same. All changes that we observe can only be changes that have already occurred, and thus “accepting this condition is nothing else but postulating the Eternal Return.”^24^ Consequently, the world of classical physics must have a symmetrical structure. The future must be identical to the past.

In chapter seven of his lectures on Hegel, Kojève points out the contradiction within this metaphysics of the real, insisting that the intellect of the classical physicist circumscribed by Meyerson corresponds to the Understanding in Hegel’s *Phenomenology*:

in pursuing its ideal of Identity, it finally leads to a universal tautology which is empty of meaning or of content, and its ‘discourse’ in the end reduces to the single word: ‘ Being’ or ‘One,’ and and so on. As soon as it wants to develop this word into genuine *discourse*, as soon as it wants to *say something*, it introduces diversity, which contradicts Identity and makes it decrepit or false from its own point of view.^25^

Last, Kojève articulates the third commitment of classical physics through a recapitulation of Laplace’s demon. In 1814, the astronomer and mathematician, Pierre-Simon Laplace, in order to explain his conception of a determinate world, had portrayed the omniscient subject at the end of the history of science:

an intellect which at a certain moment would know all forces that set nature in motion, and all positions of all items of which nature is composed, if this intellect were also vast enough to submit these data to analysis, it would embrace in a single formula the movements of the greatest bodies of the universe and those of the tiniest atom; for such an intellect nothing would be uncertain and the future just like the past could be present before its eyes.^26^

According to Kojève, while the intellect delineated by Laplace far surpasses that of the human being—it is omniscient, capable of detailed and precise predictions—this difference is only quantitative not qualitative. The world is given to them in the same way. Even though Laplace’s intellect knows the world in its totality, it is not situated outside the world, for “it does not contemplate the world *sub specie aeterni*.”^27^ Laplace’s intellect remains a “‘human being in the world,’” it has a definitive even if arbitrary place in space-time.^28^

Strictly speaking, so Kojève insists, the Laplacian intellect resembles more a point in space than a moment in time, since “in a certain sense we can therefore say that the world of classical physics is determined in relation to time but not in relation to space.”^29^ It knows “not only no change, but no time at all […] [w]hat differs ‘in time’ is analogous to what differs spatially and at most by fixing a direction with respect to the t-axis.”^30^ By spatializing time, the classical physicist reduces the subject to a geometrical point, to a system of coordinates: “[i]n this the problem of the Subject is, so to speak, concentrated, but at the same time rendered ‘harmless’: the coordinate system allows one to form and grasp the object while at the same time forgo of the subject.”^31^

This effort exemplifies a tension within the project of classical physics: the incongruity between the inevitability of postulating a subject to allow for the possibility of prediction, while simultaneously attempting to efface the subject by reducing it to a geometrical point in a coordinate system. As Kojève observes, “[i]t is not sufficient to geometrize physics, as Plato and Descartes do; it would still be necessary to geometrize the thought of the philosopher who performs this geometrization—that is, to exclude Time from this thought itself; now, this is impossible.^32^ Classical physics thus finds itself in a double bind, it can neither recognize nor annihilate the subject of science.

In *Atheism*, Kojève argues that it is precisely this attempt to efface the distinction between space and time that characterizes classical physics. Through the geometrization of the real, the intellect of the classical physicist disavows not only the possibility for change but also the finitude of the subject imposed by death:

the limitlessness of physics is expressed by the fact that there is no death: this is merely a ‘ change’ that changes nothing; i.e., nothing arises from nonbeing and nothing is transformed into nonbeing (compare the *Erhaltungssätze* [laws of conservation] of classical physics; physics knows, of course, that individuals die, but, strictly speaking, it does not know individuals).^33^

By denying the existence of the individual, the classical physicist comes to resemble the Nihilist and the silent mystic in Kojève’s reading of the *Phenomenology*. All three “conceptual characters” embody the ideal of self-annihilation—the physicist through self-effacement, the Nihilist through suicide, and the mystic through the abandonment of discourse.^34^ Although Kojève often praises the “religious Buddhist” for their attainment of “absolute silence” and regards suicide as the freest human act, in which all self-interest is annihilated, he emphasizes that it is the possibility of this act, rather than its actualization, that reveals the freedom of the human being in the world.^35^ Rather than attaining freedom through self-effacement, suicide, or abandoning discourse, it is the human being who says “I,” who accounts for their existence and risks their life in the struggle for prestige, that inaugurates history and embodies the contradictions of dialectics.

In the second chapter of his lectures on Hegel, Kojève concludes that the Nihilist (and by implication, the classical physicist and the silent mystic):

does not interest Hegel, because, by definition, he disappears by committing suicide, he ceases to be, and consequently he ceases to be a human being, an agent of historical evolution […] Only the Nihilist who *remains alive* is interesting. Now, this latter must eventually perceive the contradiction implied in his existence. And, generally speaking, the awareness of a *contradiction* is what moves human, historical evolution.^36^

## The eternal concept is related to time

In his book on Kant, Kojève identifies the Enlightenment philosopher as the first to recognize this tension within metaphysics, transforming the real of classical physics—the “Silence” of the mystic and the “Beyond of death” of the Nihilist—into a notion: the “Thing-in-itself (that is, *Eternity*).”^37^ To make discourse about the Thing-in-itself possible, Kant develops a distinctive manner of speaking, the mode of the *as if*. When we speak in this mode, we no longer affirm the reality of *that* which we speak *of*. This mode, so Kojève argues, is introduced by Kant to postulate the Thing-in-itself while still being able to close off his System of Knowledge:

Kant affirms that the ‘ Noumenon’ (= Eternity) is a Notion in the proper sense of the word, that is to say a word endowed with a sense that is not contradictory in itself and that must make part of the uni-total Discourse or of the System of Knowledge: it is, as he says, ‘inevitable.’^38^

On Kojève’s reading of the section “Of the Ultimate End of the Pure Use of Reason” of the *Critique of Pure Reason*, Kant had introduced the Thing-in-itself solely for “practical ends.”^39^ He sought to have a complete System of Knowledge *now*, rather than at *the end of history*: “[the ends of man] are everywhere and always valid where a man wants to be human, not only at the *end* of History, but from its first *beginning*.”^40^ But since neither the ends of man nor the complete System of Knowledge are realized or actualized *now*, Kant could only speak of them in the mode of the *as if*: “[t]he notion of Thing-in-itself is present in Kant’s System so that he can speak, if only in the mode of as-if, of what is, for Kant, the ‘Supreme End’ of the human in Man.”^41^

According to Kojève, Kant’s introduction of the Thing-in-itself marks a decisive advance over the classical physicist as well as the silent mystic, on the hand, and the Christian theist affirming the transcendence of the divine and thereby bringing about an unintelligible rupture within Being, on the other hand, yet the mode of *as if* still introduces a split in his System of Knowledge that generates an “irreducible lacuna”:

Kant avoids the danger of explicit or implicit contra-diction inherent in all Platonism or Theism, by expressly adopting the ‘as-if’ mode when speaking of the trans-spatio-temporal thing-in-itself. His System can thus be and remain total as a Discourse. But in doing so, it ceases to be truly unified and homogenous.^42^

It is this split in Kant’s philosophy—due to the postulation of the Thing-in-itself with what Kojève calls “subjective certainty”—that marks the introduction of dualism in philosophy and the subject into discourse.^43^

As Kojève relates in *The Idea of Determinism*, this Kantian subject became incorporated into nineteenth-century physics, when physicists began to see that the idea of the world as determinate is founded on a belief in “the indefinite extension of the sphere of explanation and causal predictions whether in the direction of the infinitely great or the infinitely small.”^44^ Classical physics, however, had never offered any demonstration of this claim.^45^ Physicists failed to present examples of the kind of predictions Laplace envisioned: “as for the possibility of predicting the future evolution of a particular phenomenon, it is easy to see that it is purely illusory.”^46^ All predictions are approximate, valid only within arbitrarily isolated systems, and specific to particular phenomena: “[t]he world taken in its totality is not the immediate object of physics.”^47^ At the end of the nineteenth-century, physicists realized that the idea of a determinate world could only be affirmed with subjective certainty.

According to Kojève, it is through the recognition of these limits that the worldview of classical physics became a fiction, understood as “a ‘regulative’ idea (in the Kantian sense).”^48^ No longer affirmed as valid in the form of a discursive truth, the idea of a determinate world became a articulated in the mode of the *as if*, attaining the same status as the ends of man in Kant’s philosophy: “the idea of *universal* determinism is only a limit-idea, a postulate [that is] not refuted but not proved either.”^49^ Classical physicists merely act *as if* the world is an isolated system, *as if* the same cause has the same effects.^50^ For Kojève, this recognition was the first indication of a heterogeneity within the worldview of classical physics, its impossibility of becoming a uni-totality without a postulate that cannot be discursively demonstrated.

In his book *Die Philosophie des Als Ob* (1911), Vaihinger had developed the regulative idea in Kant’s philosophy into a systematic approach to the philosophy of science.^51^ He had argued that scientists use fictions to schematize and order the manifold of sensation. Vaihinger distinguished between two types of fictions. On the one hand, there are semi-fictions, which represent deviations from reality by an act of abstraction. For example, when we posit the existence of atoms, we erase all diversity and conceive of Being as identity. On the other hand, there are real fictions, which are postulates that, when attempted to be demonstrated, result in contradictions. The paradigm for this kind of fiction is Kant’s concept of the Thing-in-itself.

Following Vaihinger, Kojève argues that after Kant the worldview of classical physics can only be understood as “indefinitely approachable.”^52^ Scientists who continued to believe in a determinate world were faced with what Kant calls the “‘infinite task’ of indefinite progress”—a discursive development of an idea that, in the case of physics, involves an indefinite increase in precision and the exclusion of all exceptions in order to make ever-more detailed and complete predictions.^53^ With the risk that while we indefinitely progress to a conception of the world as completely and strictly determinate, we may never be in a position to demonstrate it as an hypothesis, with the fiction continuing to lack reality, acquiring the same status as the Thing-in-itself.

By becoming a regulative idea, the worldview of classical physics came to assume an ambiguous status, oscillating between Vaihinger’s semi-fiction and real fiction: “the As-if is neither true nor even veri-similitude (as the Platonic Myth is for Plato), but neither is it false or non-verisimilitude.”^54^ On the one hand, the idea of a determinate world cannot be affirmed as a discursive truth, since there is no demonstration for the hypothesis. On the other hand, it cannot be denied or proven false, as there remains the possibility that, one day, physicists might confirm it. Those who reject the possibility of ever demonstrating the hypothesis of a determinate world endorse a Kantian fideism, treating the hypothesis as a postulate, a Thing-in-itself, only to be spoken of in the mode of the *as if*.^55^ But those who remain convinced that we will eventually demonstrate that the world is determinate, set themselves on the path from Kantianism to Hegelianism, with the idea being transformed from a regulative idea to a project, from an intention to a negating action.

As Kojève—now speaking as a Hegelian—puts it: “For us, too, the As-if = Project is neither true nor false, or, if you like, false at the beginning and true at the end. But this notion necessarily implies that of negating Action.”^56^ To realize the idea of a determinate world, we project this idea onto the present, thereby actualizing the future by negating the present—“to transform the given, turning an ideal that does not yet exist, a nothingness into a reality.”^57^ By negating the indeterminate world of the present, we can realize the ideal of our project, turn the Kantian mode of the *as if* from an article of faith into a discursive truth: “this idea can be transformed into *truth* only by negating *action*, which will destroy the World that does not correspond to the idea and will create by this very destruction the World in conformity with the ideal.”^58^

To illustrate this point, Kojève uses the example of the possibility of flying. Before the twentieth-century, flying was an impossibility, with the man claiming that he can fly being “a dreamer or a fool (or even a hypocrite) […] inevitably perish[ing] by crashing to the ground.”^59^ Whereas the man who through negation action transforms the ideal of going airborne into a reality, who “works in such a way as to realize his Project, he is a great Technician [a great Revolutionary].”^60^ The same logic pertains to the hypothesis of a determinate world. As Kojève explains, “science is born from the desire to transform the World in relation to Men; its final end is technical application.”^61^ The scientist who simply affirms the ideal of a determinate world without demonstration is a “madman,” his belief being “purely subjective.”^62^ But the scientist who discursively develops this ideal through negating action, can be listed among those “have made substantial contributions to the realization of this project, i.e., to the transformation of this erroneous As-if into truth properly so-called, [and] count (even if we do not know their names) among the great Technicians of history.”^63^

## The concept is time

In the second chapter of *The Idea of Determinism*, Kojève contends that in the late nineteenth century, most physicists had embraced this conception of the worldview of classical physics as a project. However, at the turn of the century, this view began to shift: “ since Max Planck published (in 1900) his famous memoir (on black body radiation), which is the basis of modern physics, the situation has radically changed.”^64^ For Kojève, Planck’s discovery that “physical action has a discontinuous structure, that there is an absolute minimum of action” was “revolutionary.”^65^ It indicated a limit to the possibility of indefinite progress towards complete precision, representing “a radical rupture with the classical postulate of the continuity of all physical actions.”^66^ By demonstrating that the physical world is discontinuous, Planck’s work made it impossible to distinguish between causes with different effects.

In Kojève’s view, Heisenberg’s formulation of the uncertainty principle was the inevitable sequel to Planck’s discovery.^67^ Although philosophers such as Émile Boutroux had long rejected the principle of causality, their critique remained merely “philosophical” relying on a priori arguments that were general, vague, and always contestable.^68^ In Heisenberg’s work, he discerns the first clearly articulated and detailed critique of causality within modern physics: “the critique of the principle of causality received a precise *physical* meaning.”^69^ Before 1926, it had been possible for physicists to maintain that the error in their measurements was to be effaced at the end of the infinite task, but the principle of uncertainty demonstrated that the idea of determinism as a (physical) hypothesis was no longer tenable. There were now definite limits to what could be measured and predicted. Heisenberg had uncovered the existence of uncertainty within the real.

With Heisenberg’s results, the idea of determinism as a project was falsified: “[t]here is no sense in saying that the physical systems must be considered *as if* they do not obey the principle of causality: on the contrary, the world composed of these *physical* systems is *objectively* devoid of causal structure.”^70^ The limits of causality could no longer be understood as limits imposed by the incompleteness of science. They had become definitive, for “we do not consider the world *as if* it did not have a causal structure, but the world we consider does not really have it.”^71^

While the mode of the *as if* had marked the introduction of the subject into discourse, this Kantian subject had remained completely separated from the world. It had been a mere fiction, an “ idealized general gnoseological subject, the *Bewusstsein überhaupt*.”^72^ In quantum physics, this separation between the subject and the world can no longer be upheld. The object can no longer be understood as a being-in-itself, a being independent of the thought that describes it. In his review of René Poirier’s *Essai sur quelques caractères des notions d’espace et de temps* (1932), Kojève insists that this inseparability of the subject from the object “ in quantum theory” requires the introduction of “a specifically physical subject.”^73^

As Kojève explains, an observation in quantum physics results from an interaction between an observing and an observed system. This interaction of subject and object in Heisenberg’s experiments shows that the subject of physics cannot be detached from the world or possess an ideal existence, since “interaction presupposes (or conditions, or expresses) the homogeneity of the interaction in terms of the way of their being.”^74^ Within modern physics, there is no longer a distinction between the world as a being-in-itself and the world as it appears to us: “[w]hat first of all characterizes the subject of Heisenberg’s experiments is its belonging to the same ontological region as that to which its object belongs.”^75^

Throughout *The Idea of Determinism*, Kojève follows Bohr’s interpretation in distinguishing between observing and observed system, yet insists that their complementarity comprises a uni-totality: “observing system and observed system both belong to the same ontological region and are homogeneous as to their reality and their ‘mode of being’” [*Seinsweise*].^76^ Although quantum physics introduces a dualism between observing and observed systems, this heterogeneity indicates a duality not between two Beings but within one Being, within which subject and object are equally real in relation to one another and occupy “the same ontological plane.”^77^ The real of this ontological plane or region includes both the real of classical and the real of modern physics, with the two being complementary yet different, “this real has an essentially dualistic structure, because it is the interaction of two systems, [which are] inseparable but really distinct and opposed.”^78^ In Kojève’s metaphysical terms, the Being of the modern physicist is a Totality composed of an interaction between Subject and Object.

Rather than developing Bohr’s interpretation of quantum physics along the lines of Mach’s phenomenalism, Kojève understands the complementarity of the observing and observed system to be an expression of philosophical realism:

[I]mprecision is the expression of the essential difference between the two [i.e. the observing and observed system], of the impossibility of reducing one to the other. In this sense the Heisenberg relation is the physico-mathematical expression of the fundamental thesis of realism: being is not reducible to thought; a real entity is not a realized concept, and the concept is not the entity minus its existence, but there is an essential difference between concept and reality which prevents them from completely coinciding.^79^

In his lectures on Hegel, Kojève reconceptualizes this thesis of realism as an ontological difference between the human being and the natural being. He insists that “for there to be ‘ Realism,’ the concept (knowledge) must be opposed to the thing (the object).”^80^ This dualism of the opposition between the natural and the human being, he articulates on three levels: “on the phenomenological level, *Sein* is opposed to *Selbst*; on the metaphysical level, Space to Time; on the ontological level, Identity to Negativity.”^81^ On the first level, we find the classical distinction between a (known) object and a (knowing) subject, between a being-in-itself and a being-for-itself. On the second level, there is the distinction that had exemplified the tension within the project of classical physics, that between space as a homogenous uni-totality and that of the subject of experience as time. On the third level, we have a distinction that subsumes the previous two: Being and Space are assimilated to the Identity and the Subject and Time to Negativity.

On Kojève's reading, the human being for Hegel is essentially negativity, time, and action, whereas nature is static, spatial, and self-identical. This irreducibility of man and nature, as well as the observing and observed system, constitutes the realism advanced in *Introduction to the Reading of Hegel* and *The Idea of the Determinate World*. In his review of Alfred Delp’s *Tragische Existenz* (1935), Kojève notes that this dualism in Hegel can be equated with Heidegger’s ontological dualism: “the resolute acceptance of the ontological dualism, of the essential and ontologically irreducible difference between the human-being [*Dasein*] and the natural-being” [*Vorhandensein*].^82^

In Kojève’s view, it is human being as Man, understood as “Time” or “negating action,” who opposes himself to the object, intending to have his ideals realized or actualized, who embodies the contradictions and struggles of history, giving rise to a “evolution which finally comes to the universal and homogeneous State.”^83^ In the final analysis, it is error or imprecision, rather than truth or knowledge, that compels Kojève to posit his ontological dualism—“the problem which calls for a ‘realist’ solution, therefore, is the problem of error and not of truth.”^84^ What is decisive, then, for the struggle required to institute the universal, homogenous state is the very possibility of overcoming error. It is this that will eventually result in “the overcoming of Man (that is of Time, that is, of Action).”^85^ As Kojève concludes, it is “when specifically human error is finally transformed into the truth of absolute science, [that] man ceases to exist as man, and history comes to an end.”^86^

## Conclusion

In a footnote to the *Introduction to the Reading of Hegel*, Kojève returns to his interpretation of modern physics, insisting that it has become aware of itself as a science, having become for-itself what it was in-itself: “this interpretation of science, on which Hegel insisted very much, is currently admitted by science itself […] it is expressed in mathematical form by Heisenberg’s relations of uncertainty.”^87^ The indeterminacy within the real revealed by quantum physics shows that science can never be completed, with complementarity demonstrating the impossibility of affirming that Being is One and Total: “modern physics leads in the end to this result: one cannot *speak* of the physical reality without contradictions.”^88^

To be able to affirm the end of discourse without implicating the completion of science, Kojève introduces another dualism, a distinction between scientific knowledge and philosophical truth: “[p]hysics can never attain Truth in the strong sense of the time […] there is no Truth in the domain of Physics (and of science in general).”^89^ He argues that there is, on the one hand, scientific knowledge, that is, the natural being revealed by mathematical algorithms, and on the other hand, philosophical truth expressed as perfect self-consciousness, that is, the coincidence of concept and reality at the end of history. Throughout *Introduction to the Reading of Hegel*, Kojève argues that while the latter is discursive and dialectical, the former is static and identical. Ultimately, nature or physical reality cannot be spoken of, it is “revealed to Man only by the articulated *silence* of algorithm.”^90^

Yet in *On Tyranny*, when Kojève gives us his most succinct description of the Wise Man, the distinction between knowledge and truth appears to be effaced:

[T]he Wise Man is, by definition, a man fully self-conscious. But since the Wise Man, as every man, is conditioned by the world where he lives, the perfect self-knowledge implies and presupposes an adequate knowledge of the extraneous world, *natural and human*. Wisdom, being the plenitude of self-consciousness, is therefore nothing else than omniscience.^91^

On this definition, the Wise Man seems to possess all the traits of the Laplacian intellect. They are fully self-conscious, have perfect and precise knowledge of themselves and the world and, crucially, are omniscient. Similarly, when characterizing the Wise Man in his lectures on Hegel, Kojève insists that the Wise Man has the capacity to “to answer all possible questions *in general*.”^92^ At the end of history, the Wise Man has attained absolute knowledge of both natural and human being: “[t]o be perfectly and completely *self*-conscious is to have at one’s disposal—at least virtually—an *encyclopaedic* knowledge in the full sense of the word.”^93^

This claim obscures the relation between Kojève’s affirmation of the incompleteness of (scientific) knowledge and his insistence on the possibility of absolute, philosophical wisdom. Does the Wise Man, who is omniscient, has perfect self-consciousness and adequate knowledge of the external world, no need for physics to answer all questions? Apparently, Kojève thinks they do not. They can be omniscient without having the capacity to make predictions about the future of the world. The point seems critical. At one moment, Kojève goes out of his way to press the point. In a few lines, he simply dismisses Hegel’s philosophy of nature, calling it his “basic error,” arguing that dialectics only bears on what is made by humans, that is, history.^94^

This dismissal of Hegel’s philosophy of nature, however, leaves us wondering how history and nature relate; how can we explain the emergence of the human being from the natural being? In a letter to Trần Đức Thảo, Kojève simply denies that “ the appearance of Man can be deduced a priori, like any other natural event […] This is a way of saying that Man’s act of self-creation remains an act of freedom.”^95^ But as Thảo insists, by radically separating the natural and human, their unity, as attested by the emergence of the human being from the natural being becomes unintelligible: “you define freedom by the exclusion of all intelligibility.”^96^ Ultimately, he faults Kojève not “for having separated nature and spirit, but rather for not having recognized that that separation only serves to actualize their identity […] And as you decline to find the reason for this separation in unity itself, the theologian will conclude that it derives from an incarnation.”^97^

Strikingly, Kojève comes curiously close to recognizing this problem in another footnote to his lectures on Hegel when he argues that “it is not sufficient that the *Phenomenology* be circular [complete] […] the entirety of the *Phenomenology* and the *Encyclopaedia*, must also be circular […] [n]ow, it is precisely there that the non-circularity of Hegel’s system is perfectly obvious.”^98^ Because of his monism—“the *dialecticity* of *everything* that exists”—Kojève argues that Hegel’s system can only be circular at the end of history.^99^ It is only at the beginning of history—that is, in Hegel’s *Phenomenology*—that we find a dualism of human and natural being, at the end of history—that is, in Hegel’s *Logic*—the distinction is supposed to disappear. It is for this reason that Kojève’s Hegel—*contra* Kojèv—had concluded that the end of history would coincide with the end of science: “ truth and science properly so-called are possible only at the end of time.”^100^

For Kojève, this conclusion is difficult to endorse. In his reading of Hegel, the end of history simply is the end of time; the end of humanity is the end of wisdom. As he puts it in his famous footnote in *Introduction to the Reading of Hegel*, what disappears with the human being at the end of history is not only Philosophy or the search for discursive Wisdom, but also that Wisdom itself. For in these post-historical animals, there would no longer be any “[discursive] *understanding* of the World and of self.”^101^ At the end of wisdom, the dualism of Being and Subject, Space and Time, of Identity and Negativity, is overcome and the human being annihilated by being reduced to a natural being: “*[t]rue* knowledge […] is selfless [*selbstlos*]— that is, inhuman.”^102^ This can occur only once the very possibility of error has been negated—when all ideals are fully realized or actualized. Yet what quantum physics revealed to Kojève was precisely the indeterminacy of the real, that is, the impossibility of complete and precise prediction. The end of history not being possible without the end of science, “the errors committed by men *endure indefinitely*.”^103^

## Data Availability

No datasets were generated or analysed during the current study.

## Footnotes

1 Kojève (2023, 89). All English translations from this book are mine.

2 See also Soler (2001) and Geroulanos, (2008).

3 Cf. Kojève (1968, 215). For discussion, see Stancio (Stancio, 2023).

4 Kojève (1990, 35). All English translations from this book are taken from the translation by the late Robert B. Williamson. I want to thank Benjamin Fingerhut of St. Augestine’s Press for giving me early access to this work.

5 Koyré, (1932, 318).

6 Strauss (2013, 151–152).

7 Kojève (2018a, 2018b, 15).

8 Kojève (2000, 35).

9 Kojève (1990, 47).

10 Kojève (1990, 164). Translation modified.

11 Kojève (2018b, 153).

12 Kojève (2018b, 85).

13 Kojève (1990, 54).

14 Kojève (1990, 46).

15 Kojève (1990, 139).

16 Kojève (1990, 32).

17 Kojève (1990, 44).

18 Kojève (1990, 46 & 31).

19 Meyerson (1930). For Kojève’s notes on this book, see BNF, Fonds Kojève, “Carnets sur la science,” 2:41.

20 See also Meyerson (1884).

21 Kojève (2010, 37).

22 Kojève (1990, 54).

23 Kojève (2018b, 152).

24 Kojève (1990, 68).

25 Kojève (1969, 197). See also Kojève (2010, 33–40).

26 Kojève (1990, 54).

27 Kojève (1990, 55).

28 Kojève (2018b, 152).

29 Kojève (1990, 57).

30 Kojève (2023, 74).

31 Kojève (2023, 26).

32 Kojève (1969, 127).

33 Kojève (2018b, 152).

34 Weslati (2022).

35 Kojève (1973, 47). See Love (2018, 285–287).

36 Kojève (1969, 54).

37 Kojève (1973, 50, 32 & 11). All English translations are taken from Kyle Moore’s unpublished translation of the book.

38 (Kojève 1973, 14).

39 (Kojève 1973, 29).

40 (Kojève 1973, 33).

41 (Kojève 1973, 28).

42 (Kojève 1973, 28).

43 (Kojève 1973, 42). See Stancio (2025).

44 Kojève (1990, 254).

45 Kojève (1990, 145).

46 Kojève (1990, 87).

47 Kojève (1990, 52).

48 Kojève (1990, 49 & 254).

49 Kojève (1990, 65).

50 Kojève (1990, 81–82).

51 Vaihinger (2021). For Kojève’s notes on this book, see BNF, Fonds Kojève.

52 Kojève (1990, 49).

53 Kojève (1973, 112).

54 Kojève (1973, 98).

55 Kojève (1990, 278).

56 Kojève (1973, 102).

57 Kojève (1969, 48).

58 Kojève (1969, 98).

59 Kojève (1973, 100).

60 Kojève (1973, 101).

61 Kojève (1969, 176).

62 Kojève (1969, 28).

63 Kojève (1973, 101).

64 Kojève (1990, 138).

65 Kojève (1990, 138).

66 Kojève (1990, 138).

67 Kojève (1990, 144).

68 Kojève (1990, 238).

69 Kojève (1990, 238). Translation modified.

70 Kojève (1990, 238).

71 Kojève (1990, 197–198). Emphasis added.

72 Kojève (1990, 158).

73 Koyré (1933, 16). Translation mine.

74 Kojève (2010, 46).

75 Kojève (1990, 191).

76 Kojève (1990, 276).

77 Kojève (1990, 286).

78 Kojève (1990, 284). Translation modified.

79 Kojève (1990, 194).

80 Kojève (1990, 157).

81 Kojève (1990, 157).

82 Kojève (2020, 211).

83 Kojève (1969, 139).

84 Kojève (1969, 157).

85 Kojève (1969, 156).

86 Kojève (1969, 156).

87 Kojève (1969, 177).

88 Kojève (1969, 147).

89 Kojève (1969, 178).

90 Kojève (1969, 147).

91 Kojève (2016, 160). Emphasis added.

92 Kojève (1969, 76).

93 Kojève (1969, 93 & 76). See Stancio (2023).

94 Kojève (1969, 146).

95 Muller (2009, 351).

96 Muller (2009, 353).

97 Muller (2009, 352–353).

98 Kojève (1969, 98). See Moore (2022).

99 Kojève (1969, 213).

100 Kojève (1969, 214).

101 Kojève (1969, 160).

102 Kojève (1969, 157).

103 Kojève (1969, 187: emphasis added).
